# Supplementary material for: Exosomal Biomarkers: A Comprehensive Overview of Diagnostic and Prognostic Applications in Malignant and Non-Malignant Disorders
Source: Biomolecules. 2025 Apr 15;15(4):587. doi: 10.3390/biom15040587 (PMC12024574; doi:10.3390/biom15040587)
Supplement: Supplementary file 1 [file biomolecules-15-00587-s001.zip › Table S1.pdf]

Table S1. Studies using miRNAs as the diagnostic and prognostic tools in exosomes.

Category	No.	miRNAs	Sample	Expression	Application	Ref.
Brain Cancer						
	**1**	miR-29c-3p and miR-219-5p	Serum	Decrease	Prognosis	(1)
	**2**	miR-190a	Serum	Increase	Prognosis	(1)
	**3**	miR-451, miR-711, and miR-935,	CSF	Increase	Diagnosis	(2)
	**4**	miR-21	Serum	Increase	Diagnosis	(3)
	**5**	miR-182	Tissue	Increase	Prognosis	(4)
	**6**	miR-486	Tissue	Increase	prognosis	(5)
	**7**	miR-328	Tissue	Decrease	Diagnosis and Prognosis	(6)
	**8**	miR-210	Serum	Increase	Diagnosis and Prognosis	(7)
	**9**	miR-2276-5p	Plasma	Decrease	Diagnosis and Prognosis	(8)
	**10**	miRNA-19a and miRNA-19b	Serum	Decrease	Diagnosis	(9)
	**11**	miR-155-5p	Plasma	Increase	Diagnosis and Prognosis	(10)
	**12**	miR-98-5p, miR-323-3p, miR-19b-3p	Serum	Decrease	Diagnosis	(11)
	**13**	miR-183-5p	Serum	Increase	Diagnosis	(11)
	**14**	miR-9-5, miR-21-5p, miR-1-3p	Tissue and Cell lines	Increase	Prognosis	(12)
	**15**	miR-138-5p	Tissue and Cell lines	Decrease	Diagnosis and Prognosis	(12)
	**16**	miR-29b	Serum	Decrease	Diagnosis and Prognosis	(13)
	**17**	miR-454-3p	Serum	Increase	Diagnosis and Prognosis	(14)
	**18**	miR-21, miR-222 and miR-124-3p	Serum	Increase	Prognosis	(15)
Lung tumor						
	**1**	miR-451a	Plasma	Increase	Prognosis	(16)
	**2**	miR-96	Serum	Increase	Diagnosis and Prognosis	(17)
	**3**	miR-19b-3p, miR-21-5p, miR-221-3p, miR-409-3p, miR-425-5p and miR-584-5p	Plasma	Increase	Diagnosis	(18)
	**4**	miR-126	Serum	Decrease	Diagnosis	(19)
	**5**	let-7a and miR-155	Serum	Decrease	Diagnosis	(20)
	**6**	miR-21	Serum	Decrease	Diagnosis	(21)

**7**	let-7f, miR-126-3p, miR-148b, miR-151-5p, miR-199a-3p, miR-221, miR-23b, miR-26a, miR-27b, and miR-423-3p	Serum	Increase	Diagnosis	(22)
**8**	miR-23b-3p, miR- 10b-5p, miR-21- 5p	Plasma	Increase	Prognosis	(23)
Breast Cancer					
**1**	miR-21 and mi-R1246	Plasma	Increase	Diagnosis	(24)
**2**	miR-145, miR-155 and miR-382	Serum	Increase	Diagnosis	(25)
**3**	miR-223-3p	Plasma	Increase	Diagnosis	(26)
**4**	miR-16, miR-93	Plasma	Increase	Diagnosis	(27)
**5**	miR-30	Plasma	Decrease	Prognosis	(27)
**6**	miR-188-3p, miR-500a-5p and miR-502-3p (miR-532-502 cluster)	Serum	Increase	Diagnosis	(28)
**7**	let-7b-5p, miR-122-5p, miR-151a-3p, miR-215-5p, miR-223-5p, miR-23a-3p, miR-660-5p, miR-126-5p, miR-146b-5p, miR-210-3p and miR-222-3p	Plasma	Increase	Diagnosis	(29)
**8**	miR-106a-3p, miR-106a-5p, miR-20b-5p, and miR-92a-2-5p	Plasma	Increase	Diagnosis	(30)
**9**	miR-106a-5p, miR-19b-3p, miR-20b-5p, and miR-92a-3p	Serum	Increase	Diagnosis	(30)
**10**	miR-16-5p, miR-106a-5p, miR-25-3p, miR-425-5p, and miR-93-5p, miR-20a-5p and miR-223-3p	Tissue	Increase	Diagnosis	(31)
**11**	let-7b-5p	Tissue	Decrease	Diagnosis	(31)
**12**	miR-148a	Serum	Decrease	Prognosis	(32)
**13**	miR-21 and miR-105	Serum	Increase	Diagnosis and Prognosis	(33)
Pancreatic Cancer					
**1**	miR-155 and miR-196a	Serum	Decrease	Diagnosis	(34)
**2**	miR-17-5p	Serum	Increase	Diagnosis	(34)
**3**	miR-214	Tissue	Decrease	Prognosis	(35)
**4**	miRNA-21	Serum and Pancreatic juice	Increase	Diagnosis and Prognosis	(36)
**5**	miRNA-21 and miRNA-210	Serum	Increase	Diagnosis and Prognosis	(37)
**6**	miR-10b, miR-21, miR-30c, and miR-181a	Serum	Increase	Diagnosis	(38)
**7**	miR-let7	Serum	Decrease	Diagnosis	(38)
**8**	miR-1246, miR-4644, miR-3976 and miR-4306	Serum	Increase	Diagnosis	(39)
**9**	miR-222	Plasma	Increase	Diagnosis and Prognosis	(40)

**10**	miR-4525, miR-451a and miR-21,	portal vein blood and PB	Increase	Diagnosis and Prognosis	(41)
**11**	miRNA-155	Pancreatic juice	Increase	Diagnosis and Prognosis	(42)
**12**	miR-1246, miR-3976, miR-4306, and miR-4644	Saliva and Serum	Increase	Diagnosis	(43)
Colorectal Cancer					
**1**	miR-23a and miR-301a	Serum	Increase	Diagnosis	(44)
**2**	miR-193a	Plasma	Decrease	Prognosis	(45)
**3**	let-7g	Plasma	Increase	Prognosis	(45)
**4**	miR-548c-5p	Serum	Decrease	Diagnosis	(46)
**5**	miR-92b	Plasma	Decrease	Diagnosis	(47)
**6**	miR-27a-5p	Serum	Decrease	Diagnosis	(48)
**7**	miR-224-5p, miR-548d-5p, miR-200a-3p, miR-320d, miR-200b-3p, and miR-1246	Plasma	Increase	Diagnosis	(48)
**8**	miR-6803-5p	Serum	Increase	Diagnosis and Prognosis	(49)
**9**	miR-27a and miR-130a	Plasma	Increase	Diagnosis	(50)
**10**	miR-122	Serum and cell line	Increase	Diagnosis	(51)
**11**	miR-548c-5p	Serum	Decrease	Prognosis	(51)
**12**	miR-99b-5p and miR-150-5p	Serum	Decrease	Diagnosis	(52)
Hepatocellular carcinoma					
**1**	miR-122, miR-125b, miR-145, miR-192, miR-194, miR-29a, miR-17-5p, miR-106a	Serum	Increase	Diagnosis	(53)
**2**	miR-370-3p	Serum	Decrease	Diagnosis and Prognosis	(54)
**3**	miR -196a-5p	Serum	Increase	Diagnosis and Prognosis	(54)
**4**	miR-122, miR-148a, and miR-1246	Serum	Increase	screening	(55)
**5**	miR-21-5p, miR-10b-5p, miR-221-3p, miR-223-3p	Serum	Increase	Diagnosis	(56)
**6**	miR-18a, miR-27a and miR-20b	Plasma	Increase	Prognosis	(57)
**7**	miR-125b	Serum	Decrease	Diagnosis and Prognosis	(58)
**8**	miR-29a, miR-29c, miR-133a, miR-143, miR-145, miR 192, and miR-505	Serum	Increase	Diagnosis and Prognosis	(59)
**9**	miR-21-5p and miR-144-3p	Serum	Increase	Diagnosis	(60)
**10**	miR-21-5p	Serum	Increase	Diagnosis	(61)
**11**	miR-92a-3p	Plasma	Increase	Prognosis	(62)

**12**	miR-4661-5p	Serum	Increase	Diagnosis	(63)
**13**	miR-10b-5p, miR-18a-5p, miR-215-5p, and miR-940	Serum and cell line	Increase	Diagnosis and Prognosis	(64)
**14**	miR-483-5p	Plasma and tissue	Increase	Diagnosis	(65)
**15**	miR-101 and miR-125b	Serum	Decrease	Diagnosis	(66)
**16**	RNA-224	Serum	Increase	Diagnosis and Prognosis	(67)
**17**	miR665	Serum and tissue	Increase	Diagnosis and Prognosis	(68)
**18**	miR-18a, miR-221, miR-222, miR-224	Serum	Increase	Diagnosis	(69)
**19**	miR-101, miR-106b, miR-122, miR-195	Serum	Decrease	Diagnosis	(69)
**20**	miR-122 and miR-148a	Serum	Decrease	Diagnosis and Prognosis	(70)
**21**	miR-21	Serum	Increase	Prognosis	(71)
**22**	miR-92b	Serum	Increase	Prognosis	(72)
**23**	miR-320d	Serum	Decrease	Diagnosis and Prognosis	(73)
**24**	miR-638	Serum	Decrease	Prognosis	(74)
**25**	miR-125b	Serum	Decrease	Prognosis	(75)
Thyroid cancer					
**1**	miR-146b, miR-222	Cell line	Increase	Diagnosis	(76)
**2**	miR-21-5p, miR-181a	Plasma	Increase	Diagnosis	(77)
**3**	miR-346, miR-10a-5p, miR-34a-5p	Plasma	Increase	Diagnosis	(78)
**4**	miR-376a-3p, miR-4306, miR-4433a-5p, and miR-485-3p	Plasma and serum	Increase	Diagnosis and Prognosis	(79)
**5**	miR-16-2-3p, miR-223-5p, miR-34c-5p, miR-182-5p, miR-223-3p, and miR-146b-5p	Plasma	Decrease	Diagnosis	(80)
**6**	miR-16-2-3p and miR-223-5p	Plasma	Increase	Diagnosis	(80)
Prostate cancer					
**1**	miR-21, miR-574, miR-375	Serum	Increase	Diagnosis	(81)
**2**	miR-200c-3p and miR-21-5p	Plasma	Increase	Diagnosis	(82)
**3**	miR-574-3p, miR-141-5p, and miR-21-5p	Urine	Increase	Diagnosis and Prognosis	(83)
**4**	miR-214	Urine	Decrease	Diagnosis	(84)
**5**	miR-21, miR-141, and miR-375	Urine	Increase	Diagnosis	(84)
**6**	miR-21 and miR-200c	Urine	Decrease	Diagnosis	(85)

**7**	miR-21, miR-204, miR-375	Urine	Increase	Diagnosis and Prognosis	(86)
**8**	miR-141	Serum	Increase	Diagnosis	(87)
**9**	miR-107 and miR-574-3p	Plasma and Urine	Increase	Diagnosis	(88)
**10**	miR-2909	Urine	Increase	Diagnosis	(89)
**11**	miR-196a-5p, miR-34a-5p, miR-143-3p, miR-501-3p and miR-92a-1-5p	Urine	Decrease	Diagnosis	(90)
**12**	miR-30b miR-126	Urine	Increase	Diagnosis	(91)
**13**	miR-1246	Serum	Decrease	Diagnosis	(92)
Kidney cancer					
**1**	miR-141 and miR-200b	Supernatant	Increase	Prognosis	(93)
**2**	miR-210 and miR-1233	Serum	Increase	Diagnosis	(94)
**3**	miR-204-5p	Urine	Increase	Diagnosis	(95)
**4**	miR-92a-1-5p,	Plasma	Decrease	Diagnosis	(96)
**5**	miR-149-3p, miR-424-3p	Plasma	Increase	Diagnosis	(96)
**6**	miR-15a	Plasma	Increase	Diagnosis	(97)
**7**	miR-let-7i-5p, miR-26a-1-3p, miR-615-3p	Plasma	Decrease	Prognosis	(98)
**8**	miR-210	Serum	Increase	Diagnosis	(99)
**9**	miR-224	Serum	Increase	Prognosis	(100)
Ovarian cancer					
**1**	miR-222-3p	Serum	Increase	Diagnosis	(101)
**2**	miR-200a-3p, miR-766-3p, miR-26a-5p, miR-142-3p, let-7d-5p, miR-130b-3p, miR-374a-5p, and miR-328-3p	Serum	Increase	Diagnosis	(102)
**4**	miR-1307 and miR-375	Serum	Increase	Diagnosis	(103)
**5**	miR-34a	Serum	Increase	Diagnosis and Prognosis	(104)
**6**	miR-16, miR-93, miR-126, miR-223	Serum	Decrease	Prognosis	(105)
**7**	miR-21, miR-100, miR-200b, and miR-320	Serum	Increase	Prognosis	(105)
**8**	miR-146b-5p	Serum	Increase	Prognosis	(106)
**9**	miR-93, miR-145 and miR-200c	Serum	Increase	Diagnosis	(107)
**10**	miR-373 miR-200amiR-200bmiR-200c	Serum	Increase	Diagnosis and Prognosis	(108)

**11**	miR-30a-5p	Urine	Increase	Diagnosis and Prognosis	(109)
**12**	miRNA-205	Plasma	Increase	Diagnosis and Prognosis	(110)
**13**	miR-4732-5p, miR-877-5p, miR-574-3p, let-7a-5p, let-7b-5p, let-7c-5p, and let-7f-5p	Plasma	Increase	Diagnosis	(111)
**14**	miR-1273f, miR-342-3p	Plasma	Decrease	Diagnosis	(111)
**15**	miR-1290	Serum	Increase	Diagnosis	(112)
**16**	miR-1260a, miR-7977, miR-192-5p	Plasma	Decrease	Diagnosis and Prognosis	(113)
Cervical cancer					
**2**	miR-30d-5p, let-7d-3p	Plasma	Decrease	Diagnosis	(114)
**3**	miR-196a, miR-486-5p	Plasma	Increase	Diagnosis and Prognosis	(115)
**4**	miR-125a-5p	Plasma	Decrease	Diagnosis and Prognosis	(115)
**5**	miR-221, miR-222	Tissue	Increase	Diagnosis	(116)
**6**	miR-21, miR-146a	cervicovaginal lavage	Increase	Diagnosis	(117)
**7**	miR-10b-5p, miR-34b-3p, miR-34c-5p, miR-34c-3p, miR-449b-5p, miR-200b-3p, miR-383-5p, miR-2110	Peritoneal lavage	Decrease	Diagnosis	(118)
**8**	miR-125a-5p	Plasma	Decrease	Diagnosis	(119)
**9**	miR-146a-5p, miR-15s1a-3p, miR-2110, miR-21-5p	Plasma	Increase	Diagnosis	(120)
**10**	miRNA-20a, miRNA-203, miRNA-21, miRNA-205, miRNA-218, miR-485-5	NA	Increase	Diagnosis	(121)
**11**	miR-877-3p	Tissue	Increase	Diagnosis	(122)
**12**	miR-24, miR-451, miR-125a	NA	Decrease	Diagnosis	(123)
**13**	miR-130a	Cell line	Increase	Diagnosis	(124)
**14**	miR-155	Peripheral blood and tissues	Increase	Diagnosis	(125)
**15**	hsa-miR-200c-3p	Urine	Increase	Diagnosis	(126)
Melanoma					
**1**	miR-15b-5p, miR-149-3p, miR-150-5p	Plasma	Increase	Diagnosis	(127)
**2**	miR-193a-3p, miR-524-5p	Plasma	Decrease	Diagnosis	(127)
**3**	miR-211-5p, miR-16	Serum	Increase	Diagnosis and Prognosis	(128)
**4**	miR-4487	Serum	Decrease	Diagnosis and Prognosis	(128)
**5**	miR-221	Serum	Increase	Diagnosis and Prognosis	(129)
**6**	miR-23a	Serum	Decrease	Diagnosis and Prognosis	(130)

**7**	miR-150-5p, miR-142-3p	Serum	Decrease	Diagnosis and Prognosis	(131)
**8**	miR-206	Serum	Decrease	Prognosis	(132)
**9**	miR-138	WB	Decrease	Diagnosis and Prognosis	(133)
**10**	let-7g-5p	Serum	Decrease	Prognosis	(134)
**11**	miR-495-3p miR-376c-3p miR-6730-3p	melanoma cell	Increase	Prognosis	(135)
**12**	miR-17, miR-19a, miR-21, miR-126, miR-149	melanoma cell	Increase	Prognosis	(136)
**13**	miR-532-5p miR-106b	melanoma cell	Increase	Prognosis	(137)
**14**	miR-125b	Serum	Decrease	Prognosis	(138)
**15**	miR-1180-3p	Serum	Decrease	Diagnosis and Prognosis	(139)
**16**	miR-1246, miR-185	Plasma	Increase	Prognosis	(140)
Leukemia					
**1**	miR-10b	Blood	Increase	Prognosis	(141)
**2**	miR-125b	Plasma	Increase	Prognosis	(142)
**3**	miR-21	BM	Increase	Diagnosis and Prognosis	(143)
**4**	miR-23b-5p	Blood	Decrease	Diagnosis	(144)
**5**	miR-155-5p	Serum	Increase	Diagnosis	(145)
**6**	miR-181b-5p	Plasma	Decrease	Diagnosis	(146)
**7**	miR-320d	Serum	Increase	Diagnosis and Prognosis	(147)
Lymphoma					
**1**	miR-22	Serum	Increase	Diagnosis and Prognosis	(148)
**2**	miR-485-3p	Plasma	Increase	Diagnosis	(149)
**3**	miR-375-3p, miR-107	Plasma	Decrease	Diagnosis	(149)
**4**	miR-483-3p and miR-451a	Serum	Decrease	Diagnosis and Prognosis	(150)
**5**	miR-379-5p, miR-135a-3p and miR-4476	Serum	Increase	Diagnosis and Prognosis	(150)
**6**	miR-3960, miR-6089, miR-939-5p	Plasma	Decrease	Diagnosis	(151)
**7**	miR-124, miR-532-5p	Plasma	Increase	Diagnosis	(152)
**8**	miR-425, miR-145	Plasma	Decrease	Diagnosis	(152)
**9**	miR-21-5p, miR-15a-3p	Serum	Increase	Diagnosis	(153)
**10**	miR-181a-5p, miR-210-5p	Serum	Decrease	Diagnosis	(153)

**11**	miR-125b-5p, miR-99a-5p	Serum	Increase	Prognosis	(154)

miR: *Micro* ribonucleic acid**.**

References

1. Zhi F, Zhou G, Wang S, Shi Y, Peng Y, Shao N, et al. A microRNA expression signature predicts meningioma recurrence. International journal of cancer. 2013;132(1):128-36.
2. Drusco A, Bottoni A, Lagana A, Acunzo M, Fassan M, Cascione L, et al. A differentially expressed set of microRNAs in cerebro-spinal fluid (CSF) can diagnose CNS malignancies. Oncotarget. 2015;6(25):20829.
3. Masoudi MS, Mehrabian E, Mirzaei H. MiR-21: A key player in glioblastoma pathogenesis. Journal of cellular biochemistry. 2018;119(2):1285-90.
4. Jiang L, Mao P, Song L, Wu J, Huang J, Lin C, et al. miR-182 as a prognostic marker for glioma progression and patient survival. The American journal of pathology. 2010;177(1):29-38.
5. Song L, Lin C, Gong H, Wang C, Liu L, Wu J, et al. miR-486 sustains NF-κB activity by disrupting multiple NF-κB-negative feedback loops. Cell research. 2013;23(2):274-89.
6. Yuan J, Zheng Z, Zheng Y, Lu X, Xu L, Lin L. microRNA-328 is a favorable prognostic marker in human glioma via suppressing invasive and proliferative phenotypes of malignant cells. International Journal of Neuroscience. 2016;126(2):145-53.
7. Lan F, Yue X, Xia T. Exosomal microRNA-210 is a potentially non-invasive biomarker for the diagnosis and prognosis of glioma. Oncology letters. 2020;19(3):1967-74.
8. Sun J, Sun Z, Gareev I, Yan T, Chen X, Ahmad A, et al. Exosomal miR-2276-5p in plasma is a potential diagnostic and prognostic biomarker in glioma. Frontiers in Cell and Developmental Biology. 2021;9:671202.
9. Ashraf NS, Mahjabeen I, Hussain MZ, Rizwan M, Arshad M, Mehmood A, et al. Role of exosomal miRNA-19a/19b and PTEN in brain tumor diagnosis. Future Oncology. 2023;19(22):1563-76.
10. Bao Z, Zhang N, Niu W, Mu M, Zhang X, Hu S, Niu C. Exosomal miR-155-5p derived from glioma stem-like cells promotes mesenchymal transition via targeting ACOT12. Cell death & disease. 2022;13(8):725.
11. Yang Q, Wei B, Peng C, Wang L, Li C. Identification of serum exosomal miR-98–5p, miR-183–5p, miR-323–3p and miR-19b-3p as potential biomarkers for glioblastoma patients and investigation of their mechanisms. Current Research in Translational Medicine. 2022;70(1):103315.
12. Zottel A, Šamec N, Kump A, Dall'Olio LR, Pužar Dominkuš P, Romih R, et al. Analysis of mir-9-5p, mir-124-3p, mir-21-5p, mir-138-5p, and mir-1-3p in glioblastoma cell lines and extracellular vesicles. International Journal of Molecular Sciences. 2020;21(22):8491.

13. Zhong F, Huang T, Leng J. Serum miR-29b as a novel biomarker for glioblastoma diagnosis and prognosis. International Journal of Clinical and Experimental Pathology. 2019;12(11):4106.
14. Shao N, Xue L, Wang R, Luo K, Zhi F, Lan Q. miR-454-3p is an exosomal biomarker and functions as a tumor suppressor in glioma. Molecular cancer therapeutics. 2019;18(2):459-69.
15. Santangelo A, Imbrucè P, Gardenghi B, Belli L, Agushi R, Tamanini A, et al. A microRNA signature from serum exosomes of patients with glioma as complementary diagnostic biomarker. Journal of neuro-oncology. 2018;136:51-62.
16. Kanaoka R, Iinuma H, Dejima H, Sakai T, Uehara H, Matsutani N, Kawamura M. Usefulness of plasma exosomal microRNA-451a as a noninvasive biomarker for early prediction of recurrence and prognosis of non-small cell lung cancer. Oncology. 2018;94(5):311-23.
17. Wu H, Zhou J, Mei S, Wu D, Mu Z, Chen B, et al. Circulating exosomal microRNA-96 promotes cell proliferation, migration and drug resistance by targeting LMO7. Journal of cellular and molecular medicine. 2017;21(6):1228-36.
18. Zhou X, Wen W, Shan X, Zhu W, Xu J, Guo R, et al. A six-microRNA panel in plasma was identified as a potential biomarker for lung adenocarcinoma diagnosis. Oncotarget. 2016;8(4):6513.
19. Grimolizzi F, Monaco F, Leoni F, Bracci M, Staffolani S, Bersaglieri C, et al. Exosomal miR-126 as a circulating biomarker in non-small-cell lung cancer regulating cancer progression. Scientific reports. 2017;7(1):15277.
20. Shikeeva A, Kekeeva T, Zavalishina L, Andreeva YY, Zaletaev D, Frank G. Expression of microRNA let-7a, miR-155, and miR-205 in tumor and tumor-adjacent histologically normal tissue in patients with non-small cell lung cancer. Arkhiv patologii. 2016;78(3):3-10.
21. Jin X, Chen Y, Chen H, Fei S, Chen D, Cai X, et al. Evaluation of tumor-derived exosomal miRNA as potential diagnostic biomarkers for early-stage non–small cell lung cancer using next-generation sequencing. Clinical cancer research. 2017;23(17):5311-9.
22. Zhu Y, Li T, Chen G, Yan G, Zhang X, Wan Y, et al. Identification of a serum microRNA expression signature for detection of lung cancer, involving miR-23b, miR-221, miR-148b and miR-423-3p. Lung cancer. 2017;114:6-11.
23. Liu Q, Yu Z, Yuan S, Xie W, Li C, Hu Z, et al. Circulating exosomal microRNAs as prognostic biomarkers for non-small-cell lung cancer. Oncotarget. 2016;8(8):13048.
24. Hannafon BN, Trigoso YD, Calloway CL, Zhao YD, Lum DH, Welm AL, et al. Plasma exosome microRNAs are indicative of breast cancer. Breast cancer research. 2016;18:1-14.
25. Gonzalez-Villasana V, Rashed MH, Gonzalez-Cantú Y, Bayraktar R, Menchaca-Arredondo JL, Vazquez-Guillen JM, et al. Presence of circulating miR-145, miR-155, and miR-382 in exosomes isolated from serum of breast cancer patients and healthy donors. Disease markers. 2019;2019(1):6852917.
26. Yoshikawa M, Iinuma H, Umemoto Y, Yanagisawa T, Matsumoto A, Jinno H. Exosome-encapsulated microRNA-223-3p as a minimally invasive biomarker for the early detection of invasive breast cancer. Oncology letters. 2018;15(6):9584-92.
27. Ni Q, Stevic I, Pan C, Müller V, Oliveira-Ferrer L, Pantel K, Schwarzenbach H. Different signatures of miR-16, miR-30b and miR-93 in exosomes from breast cancer and DCIS patients. Scientific reports. 2018;8(1):12974.
28. Zou X, Li M, Huang Z, Zhou X, Liu Q, Xia T, Zhu W. Circulating miR-532-502 cluster derived from chromosome X as biomarkers for diagnosis of breast cancer. Gene. 2020;722:144104.

29. Li M, Zou X, Xia T, Wang T, Liu P, Zhou X, et al. A five-miRNA panel in plasma was identified for breast cancer diagnosis. Cancer medicine. 2019;8(16):7006-17.
30. Li M, Zhou Y, Xia T, Zhou X, Huang Z, Zhang H, et al. Circulating microRNAs from the miR-106a–363 cluster on chromosome X as novel diagnostic biomarkers for breast cancer. Breast cancer research and treatment. 2018;170:257-70.
31. Zou X, Xia T, Li M, Wang T, Liu P, Zhou X, et al. MicroRNA profiling in serum: Potential signatures for breast cancer diagnosis. Cancer Biomarkers. 2021;30(1):41-53.
32. Li D, Wang J, Ma L-J, Yang H-B, Jing J-F, Jia M-M, et al. Identification of serum exosomal miR-148a as a novel prognostic biomarker for breast cancer. European Review for Medical & Pharmacological Sciences. 2020;24(13).
33. Rodríguez-Martínez A, de Miguel-Pérez D, Ortega FG, García-Puche JL, Robles-Fernández I, Exposito J, et al. Exosomal miRNA profile as complementary tool in the diagnostic and prediction of treatment response in localized breast cancer under neoadjuvant chemotherapy. Breast Cancer Research. 2019;21:1-9.
34. Que R, Ding G, Chen J, Cao L. Analysis of serum exosomal microRNAs and clinicopathologic features of patients with pancreatic adenocarcinoma. World journal of surgical oncology. 2013;11:1-9.
35. Ali S, Dubaybo H, Brand RE, Sarkar FH. Differential expression of microRNAs in tissues and plasma co-exists as a biomarker for pancreatic cancer. Journal of cancer science & therapy. 2015;7(11):336.
36. 中村聡. Pancreatic juice exosomal microRNAs as biomarkers for detection of pancreatic ductal adenocarcinoma: 九州大学; 2019.
37. Wu L, Zhou W-B, Zhou J, Wei Y, Wang H-M, Liu X-D, et al. Circulating exosomal microRNAs as novel potential detection biomarkers in pancreatic cancer. Oncology letters. 2020;20(2):1432-40.
38. Lai X, Wang M, McElyea SD, Sherman S, House M, Korc M. A microRNA signature in circulating exosomes is superior to exosomal glypican-1 levels for diagnosing pancreatic cancer. Cancer letters. 2017;393:86-93.
39. Madhavan B, Yue S, Galli U, Rana S, Gross W, Müller M, et al. Combined evaluation of a panel of protein and miRNA serum-exosome biomarkers for pancreatic cancer diagnosis increases sensitivity and specificity. International journal of cancer. 2015;136(11):2616-27.
40. Li Z, Tao Y, Wang X, Jiang P, Li J, Peng M, et al. Tumor-secreted exosomal miR-222 promotes tumor progression via regulating P27 expression and re-localization in pancreatic cancer. Cellular Physiology and Biochemistry. 2018;51(2):610-29.
41. Kawamura S, Iinuma H, Wada K, Takahashi K, Minezaki S, Kainuma M, et al. Exosome-encapsulated microRNA-4525, microRNA-451a and microRNA-21 in portal vein blood is a high-sensitive liquid biomarker for the selection of high-risk pancreatic ductal adenocarcinoma patients. Journal of Hepato-Biliary-Pancreatic Sciences. 2019;26(2):63-72.
42. Mikamori M, Yamada D, Eguchi H, Hasegawa S, Kishimoto T, Tomimaru Y, et al. MicroRNA-155 controls exosome synthesis and promotes gemcitabine resistance in pancreatic ductal adenocarcinoma. Scientific reports. 2017;7(1):42339.
43. Machida T, Tomofuji T, Maruyama T, Yoneda T, Ekuni D, Azuma T, et al. miR-1246 and miR-4644 in salivary exosome as potential biomarkers for pancreatobiliary tract cancer. Oncology reports. 2016;36(4):2375-81.
44. Karimi N, Feizi MAH, Safaralizadeh R, Hashemzadeh S, Baradaran B, Shokouhi B, Teimourian S. Serum overexpression of miR-301a and miR-23a in patients with colorectal cancer. Journal of the Chinese Medical Association. 2019;82(3):215-20.

45. Cho W-C, Kim M, Park JW, Jeong S-Y, Ku J-L. Exosomal miR-193a and let-7g accelerate cancer progression on primary colorectal cancer and paired peritoneal metastatic cancer. Translational oncology. 2021;14(2):101000.
46. Peng ZY, Gu RH, Yan B. Downregulation of exosome-encapsulated miR-548c-5p is associated with poor prognosis in colorectal cancer. Journal of cellular biochemistry. 2019;120(2):1457-63.
47. Min L, Chen L, Liu S, Yu Y, Guo Q, Li P, Zhu S. Loss of circulating exosomal miR-92b is a novel biomarker of colorectal cancer at early stage. International Journal of Medical Sciences. 2019;16(9):1231.
48. Tang Y, Zhao Y, Song X, Song X, Niu L, Xie L. Tumor-derived exosomal miRNA-320d as a biomarker for metastatic colorectal cancer. Journal of Clinical Laboratory Analysis. 2019;33(9):e23004.
49. Yan S, Jiang Y, Liang C, Cheng M, Jin C, Duan Q, et al. Exosomal miR-6803-5p as potential diagnostic and prognostic marker in colorectal cancer. Journal of Cellular Biochemistry. 2018;119(5):4113-9.
50. Liu X, Pan B, Sun L, Chen X, Zeng K, Hu X, et al. Circulating exosomal miR-27a and miR-130a act as novel diagnostic and prognostic biomarkers of colorectal cancer. Cancer Epidemiology, Biomarkers & Prevention. 2018;27(7):746-54.
51. Sun L, Liu X, Pan B, Hu X, Zhu Y, Su Y, et al. Serum exosomal miR-122 as a potential diagnostic and prognostic biomarker of colorectal cancer with liver metastasis. Journal of Cancer. 2020;11(3):630.
52. Zhao YJ, Song X, Niu L, Tang Y, Song X, Xie L. Circulating exosomal miR-150-5p and miR-99b-5p as diagnostic biomarkers for colorectal cancer. Frontiers in oncology. 2019;9:1129.
53. Xue X, Zhao Y, Wang X, Qin L, Hu R. Development and validation of serum exosomal microRNAs as diagnostic and prognostic biomarkers for hepatocellular carcinoma. Journal of cellular biochemistry. 2019;120(1):135-42.
54. Wei Y, Zhang Q, An L, Fang G, Hong D, Jiao T, et al. Serum exosomal microRNA-370-3p and microRNA-196a-5p are potential biomarkers for the diagnosis and prognosis of hepatocellular carcinoma. Folia Histochemica et Cytobiologica. 2022;60(3):215-25.
55. Wang Y, Zhang C, Zhang P, Guo G, Jiang T, Zhao X, et al. Serum exosomal micro RNA s combined with alpha-fetoprotein as diagnostic markers of hepatocellular carcinoma. Cancer Medicine. 2018;7(5):1670-9.
56. Ghosh S, Bhowmik S, Majumdar S, Goswami A, Chakraborty J, Gupta S, et al. The exosome encapsulated microRNAs as circulating diagnostic marker for hepatocellular carcinoma with low alpha-fetoprotein. International journal of cancer. 2020;147(10):2934-47.
57. Huang C, Tang S, Shen D, Li X, Liang L, Ding Y, Xu B. Circulating plasma exosomal miRNA profiles serve as potential metastasis-related biomarkers for hepatocellular carcinoma. Oncology Letters. 2021;21(2):1-.
58. Kim HS, Kim JS, Park NR, Nam H, Sung PS, Bae SH, et al. Exosomal miR-125b exerts anti-metastatic properties and predicts early metastasis of hepatocellular carcinoma. Frontiers in oncology. 2021;11:637247.
59. Lin X-J, Chong Y, Guo Z-W, Xie C, Yang X-J, Zhang Q, et al. A serum microRNA classifier for early detection of hepatocellular carcinoma: a multicentre, retrospective, longitudinal biomarker identification study with a nested case-control study. The Lancet Oncology. 2015;16(7):804-15.
60. Pu C, Huang H, Wang Z, Zou W, Lv Y, Zhou Z, et al. Extracellular vesicle-associated mir-21 and mir-144 are markedly elevated in serum of patients with hepatocellular carcinoma. Frontiers in physiology. 2018;9:930.

61. Hu Z, You L, Hu S, Yu L, Gao Y, Li L, Zhang S. Hepatocellular carcinoma cell-derived exosomal miR-21-5p promotes the polarization of tumor-related macrophages (TAMs) through SP1/XBP1 and affects the progression of hepatocellular carcinoma. International Immunopharmacology. 2024;126:111149.
62. Yang B, Feng X, Liu H, Tong R, Wu J, Li C, et al. High-metastatic cancer cells derived exosomal miR92a-3p promotes epithelial-mesenchymal transition and metastasis of low-metastatic cancer cells by regulating PTEN/Akt pathway in hepatocellular carcinoma. Oncogene. 2020;39(42):6529-43.
63. Cho HJ, Baek GO, Seo CW, Ahn HR, Sung S, Son JA, et al. Exosomal microRNA-4661-5p–based serum panel as a potential diagnostic biomarker for early-stage hepatocellular carcinoma. Cancer Medicine. 2020;9(15):5459-72.
64. Cho HJ, Eun JW, Baek GO, Seo CW, Ahn HR, Kim SS, et al. Serum exosomal microRNA, miR-10b-5p, as a potential diagnostic biomarker for early-stage hepatocellular carcinoma. Journal of Clinical Medicine. 2020;9(1):281.
65. Lin J, Lin W, Bai Y, Liao Y, Lin Q, Chen L, Wu Y. Identification of exosomal hsa-miR-483-5p as a potential biomarker for hepatocellular carcinoma via microRNA expression profiling of tumor-derived exosomes. Experimental Cell Research. 2022;417(2):113232.
66. Sun L, Xu M, Zhang G, Dong L, Wu J, Wei C, et al. Identification of Circulating Exosomal miR-101 and miR-125b Panel Act as a Potential Biomarker for Hepatocellular Carcinoma. International Journal of Genomics. 2021;2021(1):1326463.
67. Cui Y, Xu H-F, Liu M-Y, Xu Y-J, He J-C, Zhou Y, Cang S-D. Mechanism of exosomal microRNA-224 in development of hepatocellular carcinoma and its diagnostic and prognostic value. World journal of gastroenterology. 2019;25(15):1890.
68. Qu Z, Wu J, Wu J, Ji A, Qiang G, Jiang Y, et al. Exosomal miR-665 as a novel minimally invasive biomarker for hepatocellular carcinoma diagnosis and prognosis. Oncotarget. 2017;8(46):80666.
69. Sohn W, Kim J, Kang SH, Yang SR, Cho J-Y, Cho HC, et al. Serum exosomal microRNAs as novel biomarkers for hepatocellular carcinoma. Experimental & molecular medicine. 2015;47(9):e184-e.
70. Deng P, Li M, Wu Y. The Predictive Efficacy of Serum Exosomal microRNA-122 and microRNA-148a for Hepatocellular Carcinoma Based on Smart Healthcare. Journal of Healthcare Engineering. 2022;2022(1):5914541.
71. Lee H, Quek C, Silva I, Tasker A, Batten M, Rizos H, et al. Integrated molecular and immunophenotypic analysis of NK cells in anti-PD-1 treated metastatic melanoma patients. Oncoimmunology. 2019;8(2):e1537581.
72. Nakano T, Chen I-H, Wang C-C, Chen P-J, Tseng H-P, Huang K-T, et al. Circulating exosomal miR-92b: Its role for cancer immunoediting and clinical value for prediction of posttransplant hepatocellular carcinoma recurrence. American Journal of Transplantation. 2019;19(12):3250-62.
73. Li W, Ding X, Wang S, Xu L, Yin T, Han S, et al. Downregulation of serum exosomal miR-320d predicts poor prognosis in hepatocellular carcinoma. Journal of Clinical Laboratory Analysis. 2020;34(6):e23239.
74. Shi M, Jiang Y, Yang L, Yan S, Wang YG, Lu XJ. Decreased levels of serum exosomal miR-638 predict poor prognosis in hepatocellular carcinoma. Journal of cellular biochemistry. 2018;119(6):4711-6.
75. Liu W, Hu J, Zhou K, Chen F, Wang Z, Liao B, et al. Serum exosomal miR-125b is a novel prognostic marker for hepatocellular carcinoma. OncoTargets and therapy. 2017:3843-51.
76. Lee JC, Zhao J-T, Gundara J, Serpell J, Bach LA, Sidhu S. Papillary thyroid cancer–derived exosomes contain miRNA-146b and miRNA-222. Journal of Surgical Research. 2015;196(1):39-48.

77. Samsonov R, Burdakov V, Shtam T, Radzhabova Z, Vasilyev D, Tsyrlina E, et al. Plasma exosomal miR-21 and miR-181a differentiates follicular from papillary thyroid cancer. Tumor Biology. 2016;37:12011-21.
78. Wang Z, Lv J, Zou X, Huang Z, Zhang H, Liu Q, et al. A three plasma microRNA signature for papillary thyroid carcinoma diagnosis in Chinese patients. Gene. 2019;693:37-45.
79. Dai D, Tan Y, Guo L, Tang A, Zhao Y. Identification of exosomal miRNA biomarkers for diagnosis of papillary thyroid cancer by small RNA sequencing. European Journal of Endocrinology. 2020;182(1):111-21.
80. Liang M, Yu S, Tang S, Bai L, Cheng J, Gu Y, et al. A panel of plasma exosomal miRNAs as potential biomarkers for differential diagnosis of thyroid nodules. Frontiers in genetics. 2020;11:449.
81. Li M, Rai AJ, DeCastro GJ, Zeringer E, Barta T, Magdaleno S, et al. An optimized procedure for exosome isolation and analysis using serum samples: application to cancer biomarker discovery. Methods. 2015;87:26-30.
82. Endzeliņš E, Berger A, Melne V, Bajo-Santos C, Soboļevska K, Ābols A, et al. Detection of circulating miRNAs: comparative analysis of extracellular vesicle-incorporated miRNAs and cell-free miRNAs in whole plasma of prostate cancer patients. BMC cancer. 2017;17:1-13.
83. Samsonov R, Shtam T, Burdakov V, Glotov A, Tsyrlina E, Berstein L, et al. Lectin-induced agglutination method of urinary exosomes isolation followed by mi-RNA analysis: Application for prostate cancer diagnostic. The Prostate. 2016;76(1):68-79.
84. Foj L, Ferrer F, Serra M, Arévalo A, Gavagnach M, Giménez N, Filella X. Exosomal and non-exosomal urinary miRNAs in prostate cancer detection and prognosis. The Prostate. 2017;77(6):573-83.
85. Danarto R, Astuti I, Umbas R, Haryana SM. Urine miR-21-5p and miR-200c-3p as potential non-invasive biomarkers in patients with prostate cancer. Turkish Journal of Urology. 2019;46(1):26.
86. Koppers-Lalic D, Hackenberg M, De Menezes R, Misovic B, Wachalska M, Geldof A, et al. Non-invasive prostate cancer detection by measuring miRNA variants (isomiRs) in urine extracellular vesicles. Oncotarget. 2016;7(16):22566.
87. Li Z, Ma Y-Y, Wang J, Zeng X-F, Li R, Kang W, Hao X-K. Exosomal microRNA-141 is upregulated in the serum of prostate cancer patients. OncoTargets and therapy. 2015:139-48.
88. Bryant R, Pawlowski T, Catto J, Marsden G, Vessella R, Rhees B, et al. Changes in circulating microRNA levels associated with prostate cancer. British journal of cancer. 2012;106(4):768-74.
89. Wani S, Kaul D, Mavuduru R, Kakkar N, Bhatia A. Urinary-exosomal miR-2909: A novel pathognomonic trait of prostate cancer severity. Journal of Biotechnology. 2017;259:135-9.
90. Rodríguez M, Bajo-Santos C, Hessvik NP, Lorenz S, Fromm B, Berge V, et al. Identification of non-invasive miRNAs biomarkers for prostate cancer by deep sequencing analysis of urinary exosomes. Molecular cancer. 2017;16:1-6.
91. Matsuzaki K, Fujita K, Tomiyama E, Hatano K, Hayashi Y, Wang C, et al. MiR-30b-3p and miR-126-3p of urinary extracellular vesicles could be new biomarkers for prostate cancer. Translational Andrology and Urology. 2021;10(4):1918.
92. Bhagirath D, Yang TL, Bucay N, Sekhon K, Majid S, Shahryari V, et al. microRNA-1246 is an exosomal biomarker for aggressive prostate cancer. Cancer research. 2018;78(7):1833-44.
93. Saleeb R, Kim SS, Ding Q, Scorilas A, Lin S, Khella HW, et al., editors. The miR-200 family as prognostic markers in clear cell renal cell carcinoma. Urologic Oncology: Seminars and Original Investigations; 2019: Elsevier.

94. Zhang W, Ni M, Su Y, Wang H, Zhu S, Zhao A, Li G. MicroRNAs in serum exosomes as potential biomarkers in clear-cell renal cell carcinoma. European urology focus. 2018;4(3):412-9.
95. Kurahashi R, Kadomatsu T, Baba M, Hara C, Itoh H, Miyata K, et al. MicroRNA-204-5p: A novel candidate urinary biomarker of Xp11. 2 translocation renal cell carcinoma. Cancer Science. 2019;110(6):1897-908.
96. Xiao C-T, Lai W-J, Zhu W-A, Wang H. MicroRNA derived from circulating exosomes as noninvasive biomarkers for diagnosing renal cell carcinoma. OncoTargets and therapy. 2020:10765-74.
97. Li DY, Lin FF, Li GP, Zeng FC. Exosomal microRNA-15a from ACHN cells aggravates clear cell renal cell carcinoma via the BTG2/PI3K/AKT axis. The Kaohsiung journal of medical sciences. 2021;37(11):973-82.
98. Du M, Giridhar KV, Tian Y, Tschannen MR, Zhu J, Huang C-C, et al. Plasma exosomal miRNAs-based prognosis in metastatic kidney cancer. Oncotarget. 2017;8(38):63703.
99. Wang X, Wang T, Chen C, Wu Z, Bai P, Li S, et al. Serum exosomal miR-210 as a potential biomarker for clear cell renal cell carcinoma. Journal of Cellular Biochemistry. 2019;120(2):1492-502.
100. Fujii N, Hirata H, Ueno K, Mori J, Oka S, Shimizu K, et al. Extracellular miR-224 as a prognostic marker for clear cell renal cell carcinoma. Oncotarget. 2017;8(66):109877.
101. Ying X, Wu Q, Wu X, Zhu Q, Wang X, Jiang L, et al. Epithelial ovarian cancer-secreted exosomal miR-222-3p induces polarization of tumor-associated macrophages. Oncotarget. 2016;7(28):43076.
102. Alshamrani AA. Roles of microRNAs in ovarian cancer tumorigenesis: two decades later, what have we learned? Frontiers in oncology. 2020;10:1084.
103. Su YY, Sun L, Guo ZR, Li JC, Bai TT, Cai XX, et al. Upregulated expression of serum exosomal miR-375 and miR-1307 enhance the diagnostic power of CA125 for ovarian cancer. Journal of ovarian research. 2019;12:1-9.
104. Maeda K, Sasaki H, Ueda S, Miyamoto S, Terada S, Konishi H, et al. Serum exosomal microRNA-34a as a potential biomarker in epithelial ovarian cancer. Journal of ovarian research. 2020;13:1-9.
105. Pan C, Stevic I, Müller V, Ni Q, Oliveira-Ferrer L, Pantel K, Schwarzenbach H. Exosomal micro RNA s as tumor markers in epithelial ovarian cancer. Molecular oncology. 2018;12(11):1935-48.
106. Wu Q, Wu X, Ying X, Zhu Q, Wang X, Jiang L, et al. Suppression of endothelial cell migration by tumor associated macrophage-derived exosomes is reversed by epithelial ovarian cancer exosomal lncRNA. Cancer cell international. 2017;17:1-13.
107. Kim S, Choi MC, Jeong J-Y, Hwang S, Jung SG, Joo WD, et al. Serum exosomal miRNA-145 and miRNA-200c as promising biomarkers for preoperative diagnosis of ovarian carcinomas. Journal of Cancer. 2019;10(9):1958.
108. Meng X, Müller V, Milde-Langosch K, Trillsch F, Pantel K, Schwarzenbach H. Diagnostic and prognostic relevance of circulating exosomal miR-373, miR-200a, miR-200b and miR-200c in patients with epithelial ovarian cancer. Oncotarget. 2016;7(13):16923.
109. Zhou J, Gong G, Tan H, Dai F, Zhu X, Chen Y, et al. Urinary microRNA-30a-5p is a potential biomarker for ovarian serous adenocarcinoma. Oncology reports. 2015;33(6):2915-23.
110. Zhu Z, Chen Z, Wang M, Zhang M, Chen Y, Yang X, et al. Detection of plasma exosomal miRNA-205 as a biomarker for early diagnosis and an adjuvant indicator of ovarian cancer staging. Journal of Ovarian Research. 2022;15(1):27.

111. Liu J, Yoo J, Ho JY, Jung Y, Lee S, Hur SY, Choi YJ. Plasma-derived exosomal miR-4732-5p is a promising noninvasive diagnostic biomarker for epithelial ovarian cancer. Journal of ovarian research. 2021;14(1):59.
112. Jeon H, Seo SM, Kim TW, Ryu J, Kong H, Jang SH, et al. Circulating exosomal miR-1290 for diagnosis of epithelial ovarian cancer. Current Issues in Molecular Biology. 2022;44(1):288-300.
113. Chen L, Wang K, Li L, Zheng B, Zhang Q, Zhang F, et al. Plasma exosomal miR-1260a, miR-7977 and miR-192-5p as diagnostic biomarkers in epithelial ovarian cancer. Future oncology. 2022;18(26):2919-31.
114. Zheng M, Hou L, Ma Y, Zhou L, Wang F, Cheng B, et al. Exosomal let-7d-3p and miR-30d-5p as diagnostic biomarkers for non-invasive screening of cervical cancer and its precursors. Molecular cancer. 2019;18(1):1-8.
115. Cafforio P, Palmirotta R, Lovero D, Cicinelli E, Cormio G, Silvestris E, et al. Liquid biopsy in cervical cancer: hopes and pitfalls. Cancers. 2021;13(16):3968.
116. Pan Z-X, Zhang X-Y, Chen S-R, Li C-Z. Upregulated exosomal miR-221/222 promotes cervical cancer via repressing methyl-CpG-binding domain protein 2. European Review for Medical & Pharmacological Sciences. 2019;23(9).
117. Liu J, Sun H, Wang X, Yu Q, Li S, Yu X, Gong W. Increased exosomal microRNA-21 and microRNA-146a levels in the cervicovaginal lavage specimens of patients with cervical cancer. International journal of molecular sciences. 2014;15(1):758-73.
118. Roman-Canal B, Moiola CP, Gatius S, Bonnin S, Ruiz-Miró M, González E, et al. EV-associated miRNAs from peritoneal lavage are a source of biomarkers in endometrial cancer. Cancers. 2019;11(6):839.
119. Lv A, Tu Z, Huang Y, Lu W, Xie B. Circulating exosomal miR-125a-5p as a novel biomarker for cervical cancer. Oncology Letters. 2021;21(1):1-.
120. Ma G, Song G, Zou X, Shan X, Liu Q, Xia T, et al. Circulating plasma microRNA signature for the diagnosis of cervical cancer. Cancer Biomarkers. 2019;26(4):491-500.
121. Hasanzadeh M, Movahedi M, Rejali M, Maleki F, Moetamani-Ahmadi M, Seifi S, et al. The potential prognostic and therapeutic application of tissue and circulating microRNAs in cervical cancer. Journal of cellular physiology. 2019;234(2):1289-94.
122. Mendaza S, Fernández-Irigoyen J, Santamaría E, Arozarena I, Guerrero-Setas D, Zudaire T, et al. Understanding the molecular mechanism of miR-877-3p could provide potential biomarkers and therapeutic targets in squamous cell carcinoma of the cervix. Cancers. 2021;13(7):1739.
123. Sun L, Wang D, Li H, She H, Yang Y, Zhang H, Miao G. Significance of high YKL-40 expression regulated by miR-24 in cervical cancer progression and prognosis. Int J Clin Exp Pathol. 2016;9(5):5128-37.
124. Zhang J, Wu H, Li P, Zhao Y, Liu M, Tang H. NF-κB-modulated miR-130a targets TNF-α in cervical cancer cells. Journal of translational medicine. 2014;12:1-14.
125. Zhang Y, Wang Z-C, Zhang Z-S, Chen F. MicroRNA-155 regulates cervical cancer via inducing Th17/Treg imbalance. European Review for Medical & Pharmacological Sciences. 2018;22(12).
126. Srivastava A, Moxley K, Ruskin R, Dhanasekaran DN, Zhao YD, Ramesh R. A non-invasive liquid biopsy screening of urine-derived exosomes for miRNAs as biomarkers in endometrial cancer patients. The AAPS Journal. 2018;20:1-11.
127. Fogli S, Polini B, Carpi S, Pardini B, Naccarati A, Dubbini N, et al. Identification of plasma microRNAs as new potential biomarkers with high diagnostic power in human cutaneous melanoma. Tumor Biology. 2017;39(5):1010428317701646.

128. Stark MS, Klein K, Weide B, Haydu LE, Pflugfelder A, Tang YH, et al. The prognostic and predictive value of melanoma-related microRNAs using tissue and serum: a microRNA expression analysis. EBioMedicine. 2015;2(7):671-80.
129. Li P, He Q-y, Luo C-q, Qian L-y. Circulating miR-221 expression level and prognosis of cutaneous malignant melanoma. Medical science monitor: international medical journal of experimental and clinical research. 2014;20:2472.
130. Guo W, Wang H, Yang Y, Guo S, Zhang W, Liu Y, et al. Down-regulated miR-23a contributes to the metastasis of cutaneous melanoma by promoting autophagy. Theranostics. 2017;7(8):2231.
131. Tembe V, Schramm SJ, Stark MS, Patrick E, Jayaswal V, Tang YH, et al. MicroRNA and mRNA expression profiling in metastatic melanoma reveal associations with BRAF mutation and patient prognosis. Pigment cell & melanoma research. 2015;28(3):254-66.
132. Tian R, Liu T, Qiao L, Gao M, Li J. Decreased serum microRNA-206 level predicts unfavorable prognosis in patients with melanoma. International Journal of Clinical and Experimental Pathology. 2015;8(3):3097.
133. Meng F, Zhang Y, Li X, Wang J, Wang Z. Clinical significance of miR-138 in patients with malignant melanoma through targeting of PDK1 in the PI3K/AKT autophagy signaling pathway. Oncology Reports. 2017;38(3):1655-62.
134. Svedman FC, Lohcharoenkal W, Bottai M, Brage SE, Sonkoly E, Hansson J, et al. Extracellular microvesicle microRNAs as predictive biomarkers for targeted therapy in metastastic cutaneous malignant melanoma. PLoS One. 2018;13(11):e0206942.
135. Pegoraro A, De Marchi E, Ferracin M, Orioli E, Zanoni M, Bassi C, et al. P2X7 promotes metastatic spreading and triggers release of miRNA-containing exosomes and microvesicles from melanoma cells. Cell death & disease. 2021;12(12):1088.
136. Pfeffer SR, Grossmann KF, Cassidy PB, Yang CH, Fan M, Kopelovich L, et al. Detection of exosomal miRNAs in the plasma of melanoma patients. Journal of clinical medicine. 2015;4(12):2012-27.
137. Tengda L, Shuping L, Mingli G, Jie G, Yun L, Weiwei Z, Anmei D. Serum exosomal microRNAs as potent circulating biomarkers for melanoma. Melanoma Research. 2018;28(4):295-303.
138. Alegre E, Sanmamed MF, Rodriguez C, Carranza O, Martín-Algarra S, Gonzalez A. Study of circulating microRNA-125b levels in serum exosomes in advanced melanoma. Archives of Pathology and Laboratory Medicine. 2014;138(6):828-32.
139. Guo Y, Zhang X, Wang L, Li M, Shen M, Zhou Z, et al. The plasma exosomal miR-1180-3p serves as a novel potential diagnostic marker for cutaneous melanoma. Cancer cell international. 2021;21:1-15.
140. Levati L, Bassi C, Mastroeni S, Lupini L, Antonini Cappellini GC, Bonmassar L, et al. Circulating miR-1246 and miR-485-3p as promising biomarkers of clinical response and outcome in melanoma patients treated with targeted therapy. Cancers. 2022;14(15):3706.
141. Fang Z, Wang X, Wu J, Xiao R, Liu J. High serum extracellular vesicle miR-10b expression predicts poor prognosis in patients with acute myeloid leukemia. Cancer Biomarkers. 2020;27(1):1-9.
142. Jiang L, Deng T, Wang D, Xiao Y. Elevated serum exosomal miR-125b level as a potential marker for poor prognosis in intermediate-risk acute myeloid leukemia. Acta Haematologica. 2018;140(3):183-92.
143. Sun L-H, Tian D, Yang Z-C, Li J-L. Exosomal miR-21 promotes proliferation, invasion and therapy resistance of colon adenocarcinoma cells through its target PDCD4. Scientific reports. 2020;10(1):8271.
144. Tickner JA, Richard DJ, O'Byrne KJ. EV, microvesicles/microRNAs and stem cells in cancer. Exosomes, Stem Cells and MicroRNA: Aging, Cancer and Age Related Disorders. 2018:123-35.

145. Park B, Choi ME, Ryu KJ, Park C, Choi M, Yoon SE, et al. Exosomal miR-155-5p drives ibrutinib resistance in B-cell lymphoma. Experimental Cell Research. 2024;442(2):114248.
146. Hong Y, Liu K-K, Wang N-L, Xie Z-W, Chu J-H. Expression and Clinical Significance of Exosome Derived MiR-181b-5p in Children with Acute Lymphoblastic Leukemia. Zhongguo shi yan xue ye xue za zhi. 2023;31(3):643-8.
147. Gao X, Wan Z, Wei M, Dong Y, Zhao Y, Chen X, et al. Chronic myelogenous leukemia cells remodel the bone marrow niche via exosome-mediated transfer of miR-320. Theranostics. 2019;9(19):5642.
148. Rinaldi F, Marchesi F, Palombi F, Pelosi A, Di Pace AL, Sacconi A, et al. MiR-22, a serum predictor of poor outcome and therapy response in diffuse large B-cell lymphoma patients. British Journal of Haematology. 2021;195(3):399-404.
149. Liu J, Han Y, Hu S, Cai Y, Yang J, Ren S, et al. Circulating exosomal MiR-107 restrains tumorigenesis in diffuse large B-cell lymphoma by targeting 14-3-3η. Frontiers in Cell and Developmental Biology. 2021;9:667800.
150. Cao D, Cao X, Jiang Y, Xu J, Zheng Y, Kang D, Xu C. Circulating exosomal microRNAs as diagnostic and prognostic biomarkers in patients with diffuse large B-cell lymphoma. Hematological Oncology. 2022;40(2):172-80.
151. Caner V, Cetin GO, Hacioglu S, Baris IC, Tepeli E, Turk NS, et al. The miRNA content of circulating exosomes in DLBCL patients and in vitro influence of DLBCL-derived exosomes on miRNA expression of healthy B-cells from peripheral blood. Cancer Biomarkers. 2021;32(4):519-29.
152. Khare D, Goldschmidt N, Bardugo A, Gur-Wahnon D, Ben-Dov IZ, Avni B. Plasma microRNA profiling: Exploring better biomarkers for lymphoma surveillance. PLoS One. 2017;12(11):e0187722.
153. Inada K, Okoshi Y, Cho Y, Saito H, Iijima T, Hori M, Kojima H. Availability of circulating microRNAs as a biomarker for early diagnosis of diffuse large B-cell lymphoma. Open Journal of Blood Diseases. 2015;5(04):48-58.
154. Feng Y, Zhong M, Zeng S, Wang L, Liu P, Xiao X, Liu Y. Exosome-derived miRNAs as predictive biomarkers for diffuse large B-cell lymphoma chemotherapy resistance. Epigenomics. 2019;11(1):35-51.
