# Supplementary material for: Exosomal Biomarkers: A Comprehensive Overview of Diagnostic and Prognostic Applications in Malignant and Non-Malignant Disorders
Source: Biomolecules. 2025 Apr 15;15(4):587. doi: 10.3390/biom15040587 (PMC12024574; doi:10.3390/biom15040587)
Supplement: Supplementary file 1 [file biomolecules-15-00587-s001.zip › Table S2.pdf]

Table S2. Studies using proteins and non-coding RNAs as the diagnostic and prognostic tools in exosomes for malignancy disorders.

Category	No.	Exo Cargo/ Biomarker	Sample	Expression	Application	Ref.
Brain Cancer						
circRNA						
	**1**	circ_0072083	Serum	Increase	Prognosis	(1)
	**2**	circ0055202, circ0074920, circ0043722	Plasma	Increase	Prognosis	(2)
lncRNA						
	**1**	HOTAIR	Serum	Decrease	Diagnosis and Prognosis	(3)
Protein						
	**1**	hTERT	Serum	Increase	Diagnosis and Prognosis	(4)
Lung tumor						
Protein						
	**1**	LRG1	Urine	Increase	Diagnosis	(5)
	**2**	CD9, CD81, CD151, CD171, tetraspanin 8	Plasma	Increase	Diagnosis	(6)
	**3**	NY-ESO-1, EGFR, PLAP, EpCam and Alix	Plasma	Increase	Prognosis	(6)
Breast Cancer						
Protein						
	**1**	Metalloprotease ADAM10, tetraspanin CD9, HSP 70 and Annexin-1	Cell line and serum pleural effusions	Increase	Diagnosis	(7)
	**2**	CD24	Serum, ascites fluid	Increase	Diagnosis	(8)
	**3**	Del-1	Plasma	Increase	Diagnosis/monitoring	(9)
	**4**	Fibronectin	Plasma	Increase	Diagnosis	(10)
	**5**	Survivin	Serum	Increase	Diagnosis and Prognosis	(11)
	**6**	HER-2	Serum and cell line	Increase	Drug Resistance and Prognosis	(12)
	**7**	Annexin A2	Cell line	Increase	Prognosis	(13)
	**8**	GSTP-1	Cell, tissue and serum	Increase	Drug-resistant	(13)
	**9**	UCHL	Cell line	Increase	Adriamycin resistance	(14)
Hepatocellular carcinoma						

circRNA					
**1**	circ0028861	Serum	Decrease	Diagnosis	(15)
**2**	circ0051443	Plasma	Decrease	Diagnosis and Prognosis	(16)
**3**	circ0070396	Plasma	Increase	Diagnosis and Prognosis	(17)
**4**	circZFR	Serum	Increase	Diagnosis	(18)
lncRNA					
**1**	FAM72D-3	Serum	Increase	Diagnosis	(19)
**2**	EPC1-4	Serum	Decrease	Diagnosis	(19)
**3**	CRNDE	Serum	Increase	Diagnosis and Prognosis	(20)
**4**	ATB	Tissue	Increase	Prognosis	(21)
Thyroid cancer					
circRNA					
**3**	hsacirc_007293, hsacirc_031752, hsacirc_020135	Serum	Increase	Diagnosis	(22)
Protein					
**1**	Hsp27, Hsp60, Hsp90	Serum	Increase	Diagnosis and Prognosis	(23)
**2**	PD-1, PD-L1	Plasma	Increase	Diagnosis	(24)
Prostate cancer					
Protein					
**1**	PSA	Plasma	Increase	Diagnosis	(25)
**2**	CD9	Plasma	Decrease	Diagnosis and Prognosis	(26)
**3**	Survivin	Plasma	Increase	Diagnosis	(27)
**4**	EphrinA2	Serum	Increase	Diagnosis	(28)
**5**	ITGA3, ITGB1	Urine	Increase	Diagnosis	(29)
Kidney cancer					
Protein					
**1**	GSTA1, CEBPA, and PCBD1	Urine	Decrease	Diagnosis	(30)
Ovarian cancer					
circRNA					
**1**	Circ-0001068	Serum	Increase	Diagnosis	(31)

**2**	CircFoxp1	Serum	Increase	Diagnosis and Prognosis	(32)
lncRNA					
**1**	MALAT	Serum	Increase	Prognosis	(33)
Protein					
**1**	CD24 and EpCAM	Cell line	Increase	Diagnosis	(34)
**2**	Annexin A3	Plasma and cell line	Increase	Diagnosis	(35)
**3**	HSPB5, HSPB6, HSPB8	Serum and peritoneal fluid	Increase	Diagnosis	(36)
**4**	ChE, ZyE, lipoprotein lipase and collagen type V alpha 2	Cell line	Increase	Diagnosis	(37)
**5**	Claudin-4	Plasma	Increase	Diagnosis	(38)
**6**	TGF-β1, MAGE 3, and MAGE 6	Plasma	Increase	Diagnosis and Prognosis	(39)
**7**	CRABP2, SPP1, and TNFAIP6	Blood and Serum	Increase	Diagnosis	(40)
**8**	Soluble form of activated leukocyte cell adhesion molecule	Serum and ascites	Increase	Diagnosis and Prognosis	(41)
Cervical cancer					
circRNA					
**1**	hsa circ 0109046, hsa circ 0002577	Serum	Increase	Diagnosis	(42)
lncRNA					
**1**	MEG3	Plasm and tissue	Decrease	Diagnosis and Prognosis	(43)
**2**	PVT1	Serum and tissues	Increase	Diagnosis and Prognosis	(44)
**4**	HOTAIR, MALAT1, MEG3	Cervicovaginal lavage	Increase	Diagnosis	(45)
**5**	EXOC7	Serum	Increase	Diagnosis	(46)
Protein					
**1**	TF, CD144, CD14	Peripheral and uterine blood	Increase	Diagnosis	(47)
Leukemia					
lncRNA					
**1**	LINC00265 and LINC00467	Plasma	Decrease	Diagnosis	(48)
**2**	SNHG1	Plasma	Increase	Diagnosis	(48)
Protein					
**1**	CD33, CD117, CD34	Plasma	Increase	Diagnosis	(49)

**2**	CD34	Plasma	Increase	Diagnosis	(50)

circRNA: Circular RNA**; lncRN:** *Long* non-coding RNA**; HOTAIR:** HOX antisense intergenic RNA**; hTERT:** human telomerase reverse transcriptase**; LGR1:** Leucine-rich alpha-2-glycoprotein 1**; NY-ESO-1:** New York esophageal squamous cell carcinoma 1**; EGFR:** Epidermal growth factor receptor**; PALP:** Placental alkaline phosphatase**; EpCAM:** Epithelial cell adhesion molecule**; Alix:** ALG-2-interacting protein X**; ADAM10:** A Disintegrin and metalloproteinase domain-containing protein 10**; HSP:** Heat shock proteins**; Del-1:** Developmental endothelial locus-1**; HER2:** human epidermal growth factor receptor**; GSTP-1:** Glutathione S-transferase P1**; UCHL:** Ubiquitin carboxy-terminal hydrolase L1**; FAM72D:** Family With Sequence Similarity 72 Member D**; EPC-1:** Enhancer of polycomb homolog 1**; ATB:** Activated By TGF-Beta**; FNDC3B:** Fibronectin Type III Domain Containing 3B**; RASSF2:** Ras association domain-containing protein 2**; PSA:** Prostate-specific antigen**; ITGA3:** Integrin alpha-3**; GSTA1:** Glutathione S-Transferase Alpha 1; CEBPA: **CCAAT/enhancer-binding protein alpha; PCBD1:** Pterin-4-alpha-carbinolamine dehydratase**; EpCAM:** Epithelial cell adhesion molecule**; TGF-β1:** Transforming growth factor-β1**; MAGE-A3:** Melanoma-associated antigen 3**; CRABP2:** Cellular Retinoic Acid Binding *Protein* 2**; SPP1:** Osteopontin**; TNFAIP6:** tumor necrosis factor alpha induced protein 6**; sALCAM:** Activated leukocyte cell adhesion molecule**; MEG3:** Maternally Expressed 3**; PVT1:** Plasmacytoma Variant Translocation 1**; HOTAIR:** HOX Transcript Antisense RNA**; MALT1:** metastasis associated lung adenocarcinoma transcript 1**; EXOC7:** Exocyst Complex Component 7**; SNHG1:** Small nucleolar RNA host gene 1**.**

Reference

1. Ding C, Yi X, Chen X, Wu Z, You H, Chen X, et al. Warburg effect-promoted exosomal circ_0072083 releasing up-regulates NANGO expression through multiple pathways and enhances temozolomide resistance in glioma. Journal of Experimental & Clinical Cancer Research. 2021;40(1):164.
2. Xia D, Gu X. Plasmatic exosome-derived circRNAs panel act as fingerprint for glioblastoma. Aging (Albany NY). 2021;13(15):19575.
3. Tan SK, Pastori C, Penas C, Komotar RJ, Ivan ME, Wahlestedt C, Ayad NG. Serum long noncoding RNA HOTAIR as a novel diagnostic and prognostic biomarker in glioblastoma multiforme. Molecular cancer. 2018;17:1-7.
4. Uziel O, Kanner AA, Beery E, Lev S, Lahav M, Horn-Fichman S, et al. Is serum-derived exosomal hTERT transcript a marker of oncogenic activity in primary brain tumors? An exploratory study. Cancer Medicine. 2024;13(1):e6784.
5. Li Y, Zhang Y, Qiu F, Qiu Z. Proteomic identification of exosomal LRG1: a potential urinary biomarker for detecting NSCLC. Electrophoresis. 2011;32(15):1976-83.
6. Sandfeld-Paulsen B, Aggerholm-Pedersen N, Bæk R, Jakobsen K, Meldgaard P, Folkersen B, et al. Exosomal proteins as prognostic biomarkers in non-small cell lung cancer. Molecular oncology. 2016;10(10):1595-602.
7. Galindo-Hernandez O, Villegas-Comonfort S, Candanedo F, Gonzalez-Vazquez M-C, Chavez-Ocana S, Jimenez-Villanueva X, et al. Elevated concentration of microvesicles isolated from peripheral blood in breast cancer patients. Archives of medical research. 2013;44(3):208-14.
8. Rupp A-K, Rupp C, Keller S, Brase JC, Ehehalt R, Fogel M, et al. Loss of EpCAM expression in breast cancer derived serum exosomes: role of proteolytic cleavage. Gynecologic oncology. 2011;122(2):437-46.
9. Moon P-G, Lee J-E, Cho Y-E, Lee SJ, Jung JH, Chae YS, et al. Identification of developmental endothelial locus-1 on circulating extracellular vesicles as a novel biomarker for early breast cancer detection. Clinical cancer research. 2016;22(7):1757-66.
10. Moon P-G, Lee J-E, Cho Y-E, Lee SJ, Chae YS, Jung JH, et al. Fibronectin on circulating extracellular vesicles as a liquid biopsy to detect breast cancer. Oncotarget. 2016;7(26):40189.

11. Khan S, Bennit HF, Turay D, Perez M, Mirshahidi S, Yuan Y, Wall NR. Early diagnostic value of survivin and its alternative splice variants in breast cancer. BMC cancer. 2014;14:1-10.
12. Ciravolo V, Huber V, Ghedini GC, Venturelli E, Bianchi F, Campiglio M, et al. Potential role of HER2-overexpressing exosomes in countering trastuzumab-based therapy. Journal of cellular physiology. 2012;227(2):658-67.
13. Maji S, Chaudhary P, Akopova I, Nguyen PM, Hare RJ, Gryczynski I, Vishwanatha JK. Exosomal annexin II promotes angiogenesis and breast cancer metastasis. Molecular Cancer Research. 2017;15(1):93-105.
14. Ning K, Wang T, Sun X, Zhang P, Chen Y, Jin J, Hua D. UCH-L1-containing exosomes mediate chemotherapeutic resistance transfer in breast cancer. Journal of surgical oncology. 2017;115(8):932-40.
15. Wang Y, Pei L, Yue Z, Jia M, Wang H, Cao L-L. The potential of serum exosomal hsa_circ_0028861 as the novel diagnostic biomarker of HBV-derived hepatocellular cancer. Frontiers in genetics. 2021;12:703205.
16. Chen W, Quan Y, Fan S, Wang H, Liang J, Huang L, et al. Exosome-transmitted circular RNA hsa_circ_0051443 suppresses hepatocellular carcinoma progression. Cancer letters. 2020;475:119-28.
17. Lyu L, Yang W, Yao J, Wang H, Zhu J, Jin A, et al. The diagnostic value of plasma exosomal hsa_circ_0070396 for hepatocellular carcinoma. Biomarkers in medicine. 2021;15(5):359-71.
18. Zhou Y, Tang W, Zhuo H, Zhu D, Rong D, Sun J, Song J. Cancer-associated fibroblast exosomes promote chemoresistance to cisplatin in hepatocellular carcinoma through circZFR targeting signal transducers and activators of transcription (STAT3)/nuclear factor-kappa B (NF-κB) pathway. Bioengineered. 2022;13(3):4786-97.
19. Yao Z, Jia C, Tai Y, Liang H, Zhong Z, Xiong Z, et al. Serum exosomal long noncoding RNAs lnc-FAM72D-3 and lnc-EPC1-4 as diagnostic biomarkers for hepatocellular carcinoma. Aging (Albany NY). 2020;12(12):11843.
20. Huang XL, Zhang GM. Serum exosomal long noncoding RNA CRNDE level for hepatocellular carcinoma diagnosis. Journal of Clinical Laboratory Analysis. 2022;36(1).
21. Lee H, Quek C, Silva I, Tasker A, Batten M, Rizos H, et al. Integrated molecular and immunophenotypic analysis of NK cells in anti-PD-1 treated metastatic melanoma patients. Oncoimmunology. 2019;8(2):e1537581.
22. Yang C, Wei Y, Yu L, Xiao Y. Identification of altered circular RNA expression in serum exosomes from patients with papillary thyroid carcinoma by high-throughput sequencing. Medical science monitor: international medical journal of experimental and clinical research. 2019;25:2785.
23. Caruso Bavisotto C, Cipolla C, Graceffa G, Barone R, Bucchieri F, Bulone D, et al. Immunomorphological pattern of molecular chaperones in normal and pathological thyroid tissues and circulating exosomes: potential use in clinics. International journal of molecular sciences. 2019;20(18):4496.
24. Wang G, He L, Wang S, Zhang M, Li Y, Liu Q, et al. EV PD-L1 is correlated with clinical features and contributes to T cell suppression in pediatric thyroid cancer. The Journal of Clinical Endocrinology & Metabolism. 2020;105(8):e2970-e81.
25. Logozzi M, Angelini DF, Iessi E, Mizzoni D, Di Raimo R, Federici C, et al. Increased PSA expression on prostate cancer exosomes in in vitro condition and in cancer patients. Cancer letters. 2017;403:318-29.

26. Soekmadji C, Riches JD, Russell PJ, Ruelcke JE, McPherson S, Wang C, et al. Modulation of paracrine signaling by CD9 positive small extracellular vesicles mediates cellular growth of androgen deprived prostate cancer. Oncotarget. 2016;8(32):52237.
27. Khan S, Jutzy JM, Valenzuela MMA, Turay D, Aspe JR, Ashok A, et al. Plasma-derived exosomal survivin, a plausible biomarker for early detection of prostate cancer. 2012.
28. Li S, Zhao Y, Chen W, Yin L, Zhu J, Zhang H, et al. Exosomal ephrinA2 derived from serum as a potential biomarker for prostate cancer. Journal of cancer. 2018;9(15):2659.
29. Bijnsdorp IV, Geldof AA, Lavaei M, Piersma SR, van Moorselaar RJA, Jimenez CR. Exosomal ITGA3 interferes with non-cancerous prostate cell functions and is increased in urine exosomes of metastatic prostate cancer patients. Journal of extracellular vesicles. 2013;2(1):22097.
30. De Palma G, Sallustio F, Curci C, Galleggiante V, Rutigliano M, Serino G, et al. The three-gene signature in urinary extracellular vesicles from patients with clear cell renal cell carcinoma. Journal of Cancer. 2016;7(14):1960.
31. Chen L, Wang K, Li L, Zheng B, Zhang Q, Zhang F, et al. Plasma exosomal miR-1260a, miR-7977 and miR-192-5p as diagnostic biomarkers in epithelial ovarian cancer. Future oncology. 2022;18(26):2919-31.
32. Luo Y, Gui R. Circulating exosomal circFoxp1 confers cisplatin resistance in epithelial ovarian cancer cells. Journal of gynecologic oncology. 2020;31(5).
33. Qiu J-J, Lin X-J, Tang X-Y, Zheng T-T, Lin Y-Y, Hua K-Q. Exosomal metastasis-associated lung adenocarcinoma transcript 1 promotes angiogenesis and predicts poor prognosis in epithelial ovarian cancer. International journal of biological sciences. 2018;14(14):1960.
34. Runz S, Keller S, Rupp C, Stoeck A, Issa Y, Koensgen D, et al. Malignant ascites-derived exosomes of ovarian carcinoma patients contain CD24 and EpCAM. Gynecologic oncology. 2007;107(3):563-71.
35. Yin J, Yan X, Yao X, Zhang Y, Shan Y, Mao N, et al. Secretion of annexin A3 from ovarian cancer cells and its association with platinum resistance in ovarian cancer patients. Journal of cellular and molecular medicine. 2012;16(2):337-48.
36. Wyciszkiewicz A, Kalinowska-Łyszczarz A, Nowakowski B, Kaźmierczak K, Osztynowicz K, Michalak S. Expression of small heat shock proteins in exosomes from patients with gynecologic cancers. Scientific reports. 2019;9(1):9817.
37. Cheng L, Zhang K, Qing Y, Li D, Cui M, Jin P, Xu T. Proteomic and lipidomic analysis of exosomes derived from ovarian cancer cells and ovarian surface epithelial cells. Journal of ovarian research. 2020;13:1-13.
38. Li J, Sherman-Baust CA, Tsai-Turton M, Bristow RE, Roden RB, Morin PJ. Claudin-containing exosomes in the peripheral circulation of women with ovarian cancer. BMC cancer. 2009;9:1-11.
39. Szajnik M, Derbis M, Lach M, Patalas P, Michalak M, Drzewiecka H, et al. Exosomes in plasma of patients with ovarian carcinoma: potential biomarkers of tumor progression and response to therapy. Gynecology & obstetrics (Sunnyvale, Calif). 2013:003.
40. Li N, Lin G, Zhang Y, Zhang Q, Zhang H. Exosome-related protein CRABP2 is upregulated in ovarian carcinoma and enhances cell proliferation. Discover Oncology. 2022;13(1):33.
41. Carbotti G, Orengo AM, Mezzanzanica D, Bagnoli M, Brizzolara A, Emionite L, et al. Activated leukocyte cell adhesion molecule soluble form: a potential biomarker of epithelial ovarian cancer is increased in type II tumors. International journal of cancer. 2013;132(11):2597-605.
42. Xu H, Gong Z, Shen Y, Fang Y, Zhong S. Circular RNA expression in extracellular vesicles isolated from serum of patients with endometrial cancer. Epigenomics. 2018;10(2):187-97.

43. Zhang J, Yao T, Lin Z, Gao Y. Aberrant methylation of MEG3 functions as a potential plasma-based biomarker for cervical cancer. Scientific reports. 2017;7(1):6271.
44. Wang X, Wang G, Zhang L, Cong J, Hou J, Liu C. LncRNA PVT1 promotes the growth of HPV positive and negative cervical squamous cell carcinoma by inhibiting TGF-β1. Cancer cell international. 2018;18:1-8.
45. Zhang J, Liu SC, Luo XH, Tao GX, Guan M, Yuan H, Hu DK. Exosomal Long noncoding RNA s are differentially expressed in the Cervicovaginal lavage samples of cervical cancer patients. Journal of clinical laboratory analysis. 2016;30(6):1116-21.
46. Guo Y, Wang X, Wang K, He Y. Appraising the Value of Serum and Serum-Derived Exosomal LncRNA-EXOC7 as a Promising Biomarker in Cervical Cancer. Clinical Laboratory. 2020(7).
47. Dziechciowski M, Zapala B, Skotniczny K, Gawlik K, Pawlica-Gosiewska D, Piwowar M, et al. Diagnostic and prognostic relevance of microparticles in peripheral and uterine blood of patients with endometrial cancer. Ginekologia Polska. 2018;89(12):682-7.
48. Xiao Q, Lin C, Peng M, Ren J, Jing Y, Lei L, et al. Circulating plasma exosomal long non-coding RNAs LINC00265, LINC00467, UCA1, and SNHG1 as biomarkers for diagnosis and treatment monitoring of acute myeloid leukemia. Frontiers in Oncology. 2022;12:1033143.
49. Szczepanski MJ, Szajnik M, Welsh A, Whiteside TL, Boyiadzis M. Blast-derived microvesicles in sera from patients with acute myeloid leukemia suppress natural killer cell function via membrane-associated transforming growth factor-β1. Haematologica. 2011;96(9):1302.
50. Hong CS, Muller L, Boyiadzis M, Whiteside TL. Isolation and characterization of CD34+ blast-derived exosomes in acute myeloid leukemia. PloS one. 2014;9(8):e103310.
